# Reactive Histiocytic Proliferation in Rheumatoid Arthritis Mimicking Erdheim-Chester Disease

**DOI:** 10.7759/cureus.90818

**Published:** 2025-08-23

**Authors:** Syed Mashood Iqbal, Abrar Ali Mohammed, Mudasir R Ghani, Nadeem Maddekar, Awab Ismail

**Affiliations:** 1 Acute and General Medicine, Queen Elizabeth Hospital Birmingham, Birmingham, GBR; 2 Internal Medicine, Queen Elizabeth Hospital Birmingham, Birmingham, GBR; 3 Internal Medicine, Queen Elizabeth Hospital Birmingham, Birmingham, IND; 4 Respiratory Medicine, Queen Elizabeth Hospital Birmingham, Birmingham, GBR

**Keywords:** autoimmune inflammation, erdheim chester disease, histiocytic proliferation, immunohistochemistry, non langerhans histiocytosis, reactive histiocytosis, rheumatoid arthritis, systemic histiocytosis

## Abstract

Erdheim-Chester disease (ECD) is a rare non-Langerhans cell histiocytosis that can mimic inflammatory and neoplastic conditions, complicating diagnosis. We present a case in which ECD was initially suspected due to histiocytic proliferation but was ultimately diagnosed as rheumatoid arthritis (RA) with reactive histiocytosis.

An adult female with a 50-year smoking history presented with polyarticular joint pain, dyspnoea, and peripheral oedema. Chest imaging revealed a left pleural effusion, confirmed as an exudate (protein 39 g/L) with negative cytology. Fluorodeoxyglucose positron emission tomography-computed tomography (FDG PET-CT) showed a large joint uptake, suggestive of inflammatory arthropathy and bilateral pleural effusions without focal pulmonary lesions. Thoracoscopy and pleural biopsy revealed histiocytic proliferation, raising suspicion for ECD. Clinical examination and serology (rheumatoid factor >200 Units U/mL and anti-cyclic citrullinated peptide >340 U/mL) confirmed RA. Genetic testing of the biopsy showed no mutations, suggesting reactive histiocytosis. Methotrexate treatment for RA led to symptomatic improvement, and repeat PET-CT was stable. Haematology follow-up ruled out malignancy, discharging the patient from the hospital.

This case highlights the diagnostic challenge of distinguishing ECD from RA when histiocytic proliferation and joint involvement coexist. Genetic testing and multidisciplinary evaluation are crucial for accurate diagnosis and management.

## Introduction

As defined in a journal in the American Society of Hematology [1], Erdheim-Chester disease (ECD), though rare, poses significant diagnostic challenges due to its ability to mimic more common inflammatory and neoplastic conditions. ECD is a rare clonal non-Langerhans cell histiocytosis characterised by the infiltration of tissues by lipid-laden, foamy histiocytes that are CD68-positive and CD1a-negative. As outlined in a comprehensive study by Pegoraro et al. [2], which reports some common mutations and delves into the pathophysiology, the disease is driven, in most cases, by somatic mutations activating the MAPK pathway, most commonly the BRAF V600E mutation, as well as MAP2K1 and other related genes. These are further highlighted in a recent study by Kulkarni et al. [3], which also explores some recent advances in treatment modalities.

Clinically, ECD is highly heterogeneous, with manifestations ranging from indolent, localised disease to severe, multisystem involvement. This wide variety of presentations is expressed in a comprehensive review article exploring the differing presentations by Ozkaya et al. [4].

Diagnosis requires histopathologic confirmation of characteristic histiocytes in tissue biopsy, supported by clinical and radiologic findings. As highlighted in a study by Starkebaum and Hendrie [5], the identification of MAPK somatic mutations can help differentiate the condition from other histiocytic neoplastic conditions. Immunohistochemistry typically shows CD68+, CD1a-, and S100- histiocytes. Molecular testing for BRAF and other MAPK pathway mutations is now standard, as it guides therapy [5].

Benson et al. explored some of the common clinical presentations and radiological features on imaging [6]. The most frequent features include symmetric osteosclerosis of the long bones (especially lower limbs), retroperitoneal fibrosis ("hairy kidney" on imaging), periaortic fibrosis ("coated aorta"), cardiac pseudotumor, central nervous system (CNS) involvement (notably diabetes insipidus and cerebellar dysfunction), and, less commonly, pulmonary, cutaneous, or orbital disease. Multiorgan involvement is common and can be life-threatening, particularly with cardiac or CNS infiltration [6].

Treatment is tailored to disease severity and mutation status. Interferon-α has historically been the first-line treatment, but BRAF and MEK inhibitors are now preferred for patients with severe or refractory disease and relevant mutations, given their robust efficacy. Other options include anti-cytokine therapies (e.g., IL-1 and TNF-α blockers) and immunosuppressants in selected cases. These different treatment modalities are explored by Campochiaro et al. [7].

## Case presentation

**Figure 1:**
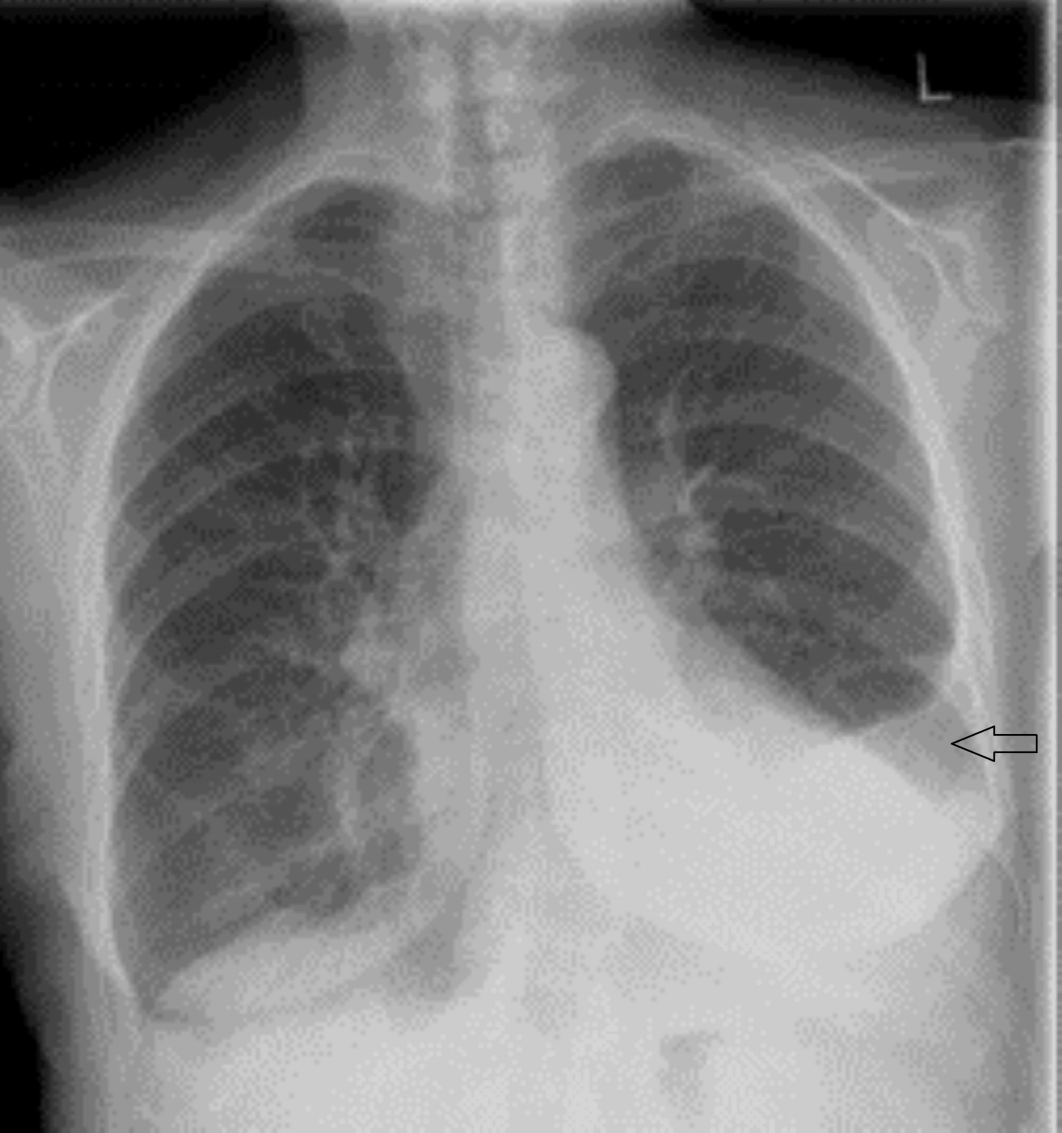
Chest X-ray (posteroanterior view) showing a moderate-sized left-sided pleural effusion.

A 73-year-old female patient with a 50-year history of smoking (15-20 cigarettes/day) presented to primary care with a three-year history of progressive joint pain affecting the knees, hands, wrists, elbows, shoulders, and ankles, worsening over the past eight months. She reported morning stiffness, limiting daily activities, along with increasing breathlessness (Medical Research Council grade 2) and bilateral leg oedema. She had intentionally lost weight but denied red flag symptoms such as rashes, Raynaud’s, or neurological complaints. A chest X-ray (Figure 1) revealed a left-sided pleural effusion, prompting further evaluation.

**Figure 2:**
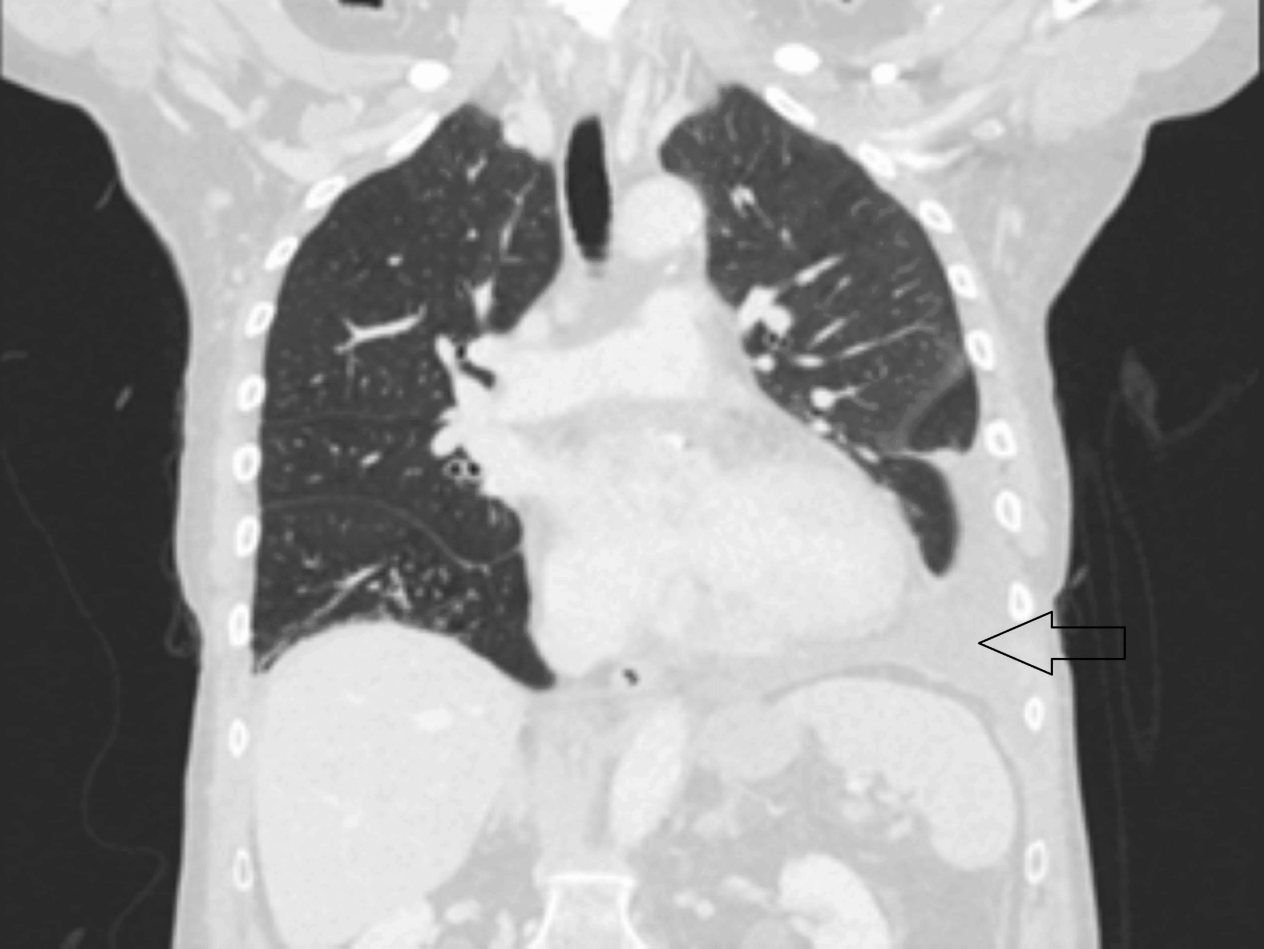
CT scan of the thorax and abdomen with contrast (coronal view) showing a moderate-sized left-sided pleural effusion with subtle pleural thickening.

Computed tomography (CT) scan of the thorax and abdomen confirmed a left pleural effusion with mild pleural thickening and a small right-sided effusion (Figure 2), initially attributed to infection, based on mildly raised infection markers, unilateral effusion, and associated atelectasis.

No pulmonary nodules or mediastinal lymphadenopathy were noted. Ultrasound-guided left-sided pleural aspiration revealed an exudate (protein 39 g/L) with negative microbiology and cytology. Due to diagnostic uncertainty, medical thoracoscopy was performed, and pleural biopsy showed histiocytic proliferation, raising suspicion for ECD, a rare non-Langerhans cell histiocytosis. The patient was referred to haematology for further management.

**Figure 3:**
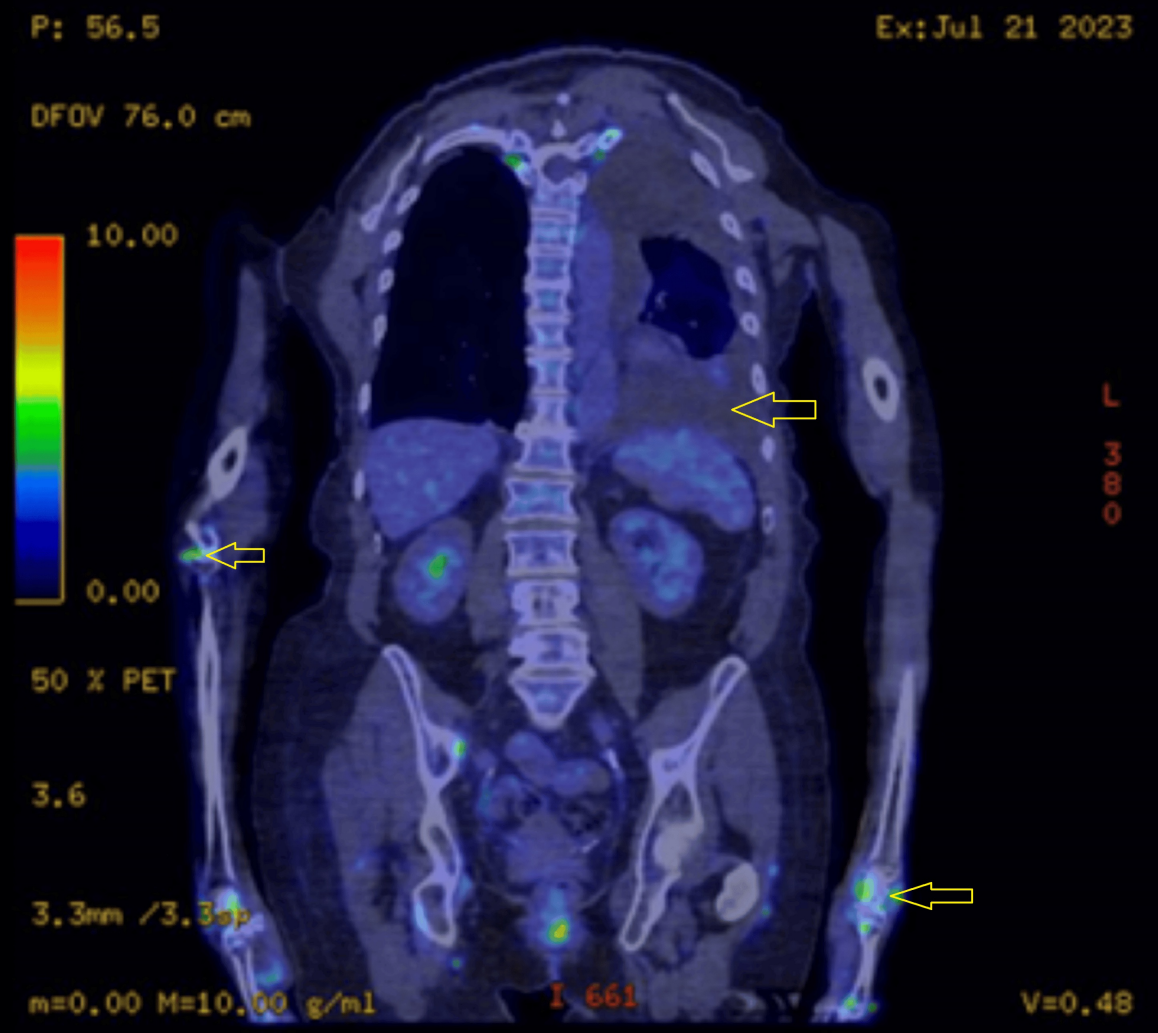
FDG PET-CT scan (coronal view) showing avid uptake in the joints with sparing of the spine and hips, and a large left pleural effusion with associated lung collapse.

A whole-body FDG PET-CT (fluorodeoxyglucose positron emission tomography-computed tomography) (Figure 3) performed in July 2023 demonstrated patchy increased tracer uptake in large joints (sparing the hips), consistent with symmetric arthropathy involving medium to large joints, possibly due to an underlying inflammatory process. It also showed a large left pleural effusion with partial left lower lobe collapse and a trace right pleural effusion, all without focal FDG avidity.

**Figure 4:**
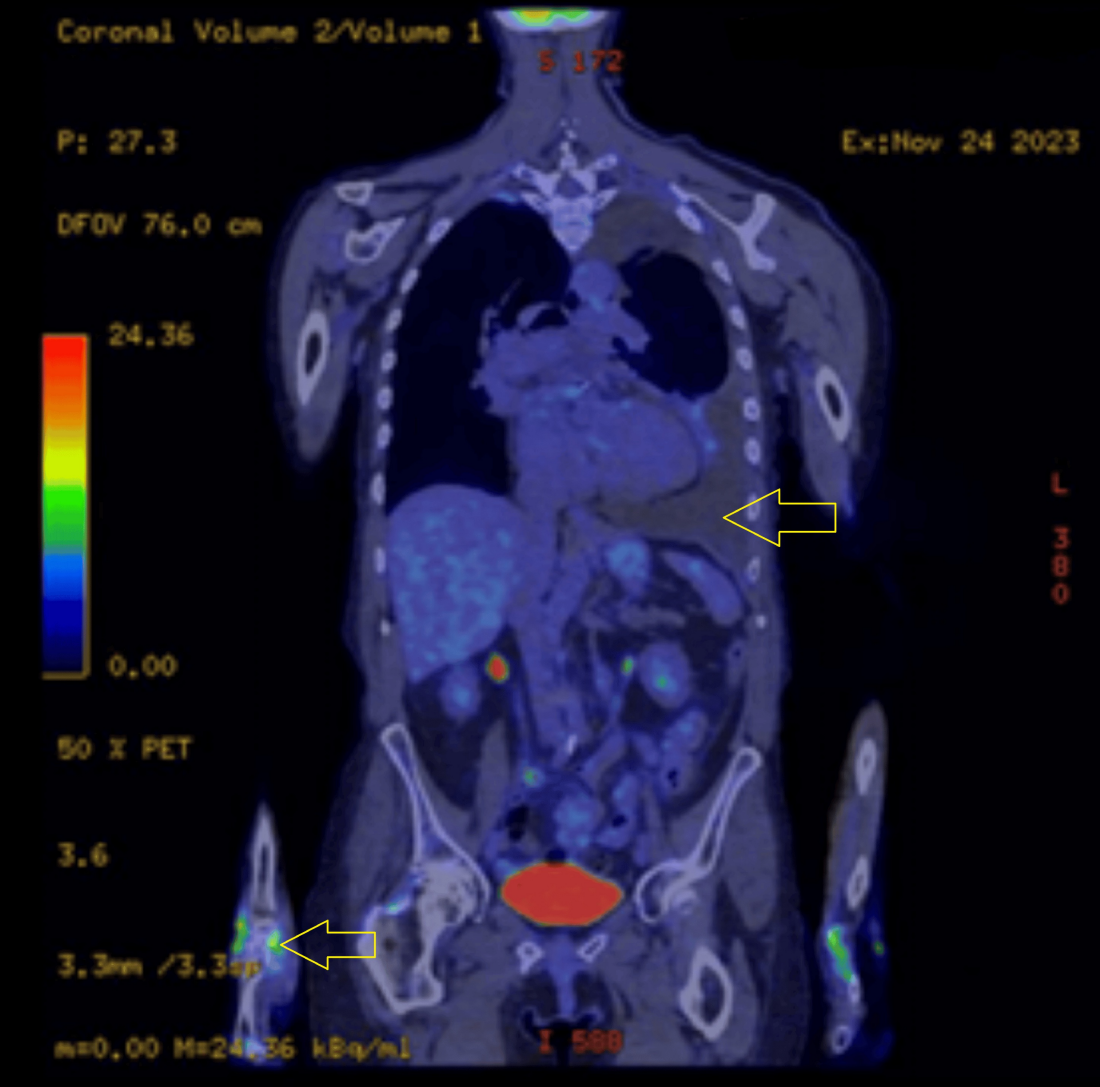
FDG PET-CT scan (coronal view) showing worsening left-sided effusion and persistent increased uptake in multiple joints.

A repeat PET-CT performed in November 2023 (Figure 4) showed mildly progressive bilateral pleural effusions (left effusion measuring 4.9 cm in depth), increased by 0.5 cm on coronal view, but no new FDG-avid lesions.

In the haematology clinic, the patient exhibited clinical signs suggestive of RA, including an elbow nodule. Rheumatology evaluation confirmed a diagnosis of RA based on swollen metacarpophalangeal (MCP) joints, tender first MCP joints, bony growths on distal interphalangeal (DIP) joints, soft elbow swellings, painful shoulder movement, and bilateral leg oedema with lymphedema. Musculoskeletal ultrasound revealed active synovitis in the hands and wrists. Serological tests showed increased rheumatoid factor (>200 U/mL) and anti-CCP (>340 U/mL), as well as raised inflammatory markers with a C-reactive protein (CRP) level of 22 mg/L (Table 1), an erythrocyte sedimentation rate (ESR) of 56 millimetres per hour (mm/hr), and an undetectable antinuclear antigen assay, complements, and other autoimmune markers. As per the ACR/EULAR RA classification criteria, the patient scored 7 points, consistent with a diagnosis of definite rheumatoid arthritis (RA).​​​​​​​

**Table 1:** Serological and inflammatory markers supporting the diagnosis of seropositive rheumatoid arthritis in a patient with reactive histiocytic pleural disease.

Laboratory Value	Patient Value	Normal Range	Units
C-reactive protein	22	1-5	Milligrams per millilitre (mg/mL)
Erythrocyte sedimentation rate	56	2-19	millimetre per hour (mm/hr)
Rheumatoid factor	>200 (above lab detectable range)	0-29.9	Unit per millilitre (U/mL)
Anti-cyclic citrullinated peptide	>340 (above lab detectable range)	0.4-6.9	Unit per millilitre (U/mL)

The patient was started on methotrexate for RA, with regular analgesia continued. She reported concentrated orange urine, but urine dipstick and culture were unremarkable. This was attributed to possible dehydration, as renal and liver functions remained stable. Genetic analysis of the pleural biopsy revealed no mutations, including the absence of BRAF V600E, suggesting that the histiocytic proliferation was reactive rather than neoplastic. Histopathological examination showed sheets of histiocytes without atypia or foamy cytoplasm, and immunohistochemistry revealed no evidence of markers typically associated with ECD. Methotrexate led to significant improvement in joint pain and mobility.

Follow-up in early 2025 showed continued clinical improvement, despite a personal loss. A repeat PET-CT was stable, with no evidence of malignancy, and the previously noted pleural effusion had resolved on imaging. The haematology team discharged the patient, concluding that the histiocytic proliferation was likely secondary to RA. Rheumatology continued to manage her RA in the escalator clinic.​​​​​​​

## Discussion

ECD is a rare non-Langerhans cell histiocytosis, with fewer than 1,000 cases reported globally. It typically affects adults between 40 and 70 years of age, with a slight male predominance. The prevalence and distribution of cases have been explored in a literature review by Mazor et al. [8], and the patient in this case aligned within the normal age range expected. Its varying presentations were explored by Ozkaya et al. [4], as it often presents as a multisystemic disease with variable organ involvement, including the skeleton, cardiovascular system, retroperitoneum, lungs, orbits, and CNS. Due to its rarity and overlapping features with other systemic diseases, ECD often poses significant diagnostic challenges.

In comparison, a case report by Sella et al. described confirmed ECD with respiratory and osseous involvement, diagnosed following a VATS procedure and biopsy [9]. Similarly, our case required thoracoscopy and histopathological analysis with immunohistochemistry to reach a definitive diagnosis. However, unlike the case in Sella et al.’s report, our patient exhibited prominent polyarthropathy, which is not typically associated with ECD and may reflect an underlying inflammatory arthritis. Additionally, systemic lupus erythematosus (SLE) was considered as a differential diagnosis prior to thoracoscopy; however, serological testing including double-stranded DNA, antinuclear antibodies, and extractable nuclear antigens was negative, effectively excluding SLE.

In terms of imaging appearance in cases of ECD, a comprehensive retrospective study by Brun et al. reviewed the distribution of radiological appearances. In that particular study, lung involvement was demonstrated in 55% of cases, and, in most cases, it involved pleural thickening and effusion, similar to our case [10].

In this case, a middle-aged female with a significant smoking history presented with polyarticular joint pain, dyspnoea, and peripheral oedema. Initial evaluation revealed bilateral pleural effusions, and FDG-PET/CT showed increased uptake in large joints, which raised the possibility of inflammatory arthropathy. Histological analysis of pleural biopsies revealed histiocytic proliferation, a key feature of ECD, further prompting consideration of this diagnosis. However, serological findings (RA factor >200 IU/mL, anti-CCP >340 U/mL) and clinical presentation were consistent with seropositive RA, a more common autoimmune condition known to involve both joints and the pleura. On review, this patient’s presentation with evidence of skeletal and possibly pulmonary involvement also aligned with what has been observed in the literature on ECD.

Differential diagnoses in this context include Langerhans cell histiocytosis (LCH), IgG4-related disease, and sarcoidosis. LCH was excluded by immunophenotyping, as histiocytes lacked CD1a and Langerin expression. IgG4-related disease typically involves elevated serum IgG4 levels and tissue infiltration by IgG4-positive plasma cells; in this case, serum IgG4 was within normal range (0.02 g/L), and no IgG4-positive plasma cells were identified histologically. Sarcoidosis was ruled out due to the absence of non-caseating granulomas, pulmonary nodules, or hilar lymphadenopathy on imaging. Para-neoplastic conditions, which may mimic histiocytic disorders, have been highlighted by Haroche et al. [1]. Histological analysis of the left pleural biopsy revealed histiocytic proliferation without atypia. Immunohistochemistry showed CD68-positive and CD1a-negative cells, and BRAF mutation testing revealed no evidence of V600E mutation.

Although the histological findings raised suspicion for ECD, there were many key differences when compared to classical cases of ECD in the literature. One difference is the absence of skeletal radiologic hallmarks, such as symmetric diaphyseal osteosclerosis, which was described by Benson et al. as a hallmark feature of ECD [6]. Another key difference is the lack of systemic organ involvement beyond the pleura, and negative molecular testing for BRAF^V600E and other MAPK, which are genetic pathway mutations highly suggestive of the diagnosis, as outlined by Ozkaya et al. [4]. Genetic profiling plays a critical role in differentiating true clonal histiocytic neoplasms such as ECD from reactive histiocytosis, as seen in autoimmune or inflammatory settings. In this patient, the lack of detectable mutations favoured a reactive process associated with underlying RA.

## Conclusions

Our patient responded well to methotrexate, a conventional disease-modifying antirheumatic drug, which further supported a diagnosis of RA with reactive histiocytic features. Follow-up PET-CT demonstrated stability, and haematology review excluded an underlying malignancy.

This case illustrates the complex interplay between autoimmune and histiocytic diseases and the diagnostic pitfalls that can arise when histiocytic proliferation occurs in a background of systemic inflammatory disease. It underscores the need for a multidisciplinary approach that synthesises clinical, imaging, pathological, and molecular data to ensure diagnostic accuracy. Accurate differentiation between ECD and reactive histiocytosis is essential, as treatment strategies and prognoses differ significantly. A structured diagnostic approach is recommended, beginning with early laboratory investigations for autoimmune and inflammatory markers, followed by cross-sectional imaging to screen for malignancy, nuclear medicine imaging to identify metabolically active lesions, and histopathological examination that includes immunohistochemistry and genetic mutation analysis to guide definitive diagnosis and management.
